# Pharmacokinetics and pharmacodynamics of the factor XIa‐inhibiting antibody osocimab in healthy male East Asian volunteers: Results from two phase 1 studies

**DOI:** 10.1002/prp2.70012

**Published:** 2024-09-22

**Authors:** Zhili Dong, Kensei Hashizume, Frauke Friedrichs, Pei Liu, Toshiaki Tanaka, Yuqin Liao

**Affiliations:** ^1^ Clinical Pharmacology Asia Bayer Healthcare Company Ltd Shanghai China; ^2^ Clinical Pharmacology, Translational Sciences, Research & Development Japan Bayer Yakuhin, Ltd Tokyo Japan; ^3^ Research & Early Development Statistics, Bayer AG Wuppertal Germany; ^4^ Clinical Pharmacology Asia Bayer Healthcare Company Ltd Beijing China; ^5^ Clinical Statistics & Analytics China Bayer Healthcare Company Ltd Beijing China

**Keywords:** cardiovascular pharmacology, pharmacokinetics, phase I

## Abstract

The pharmacokinetics, pharmacodynamics, immunogenicity, and safety of osocimab single doses in healthy Chinese and Japanese volunteers over 149 days were evaluated. Two phase 1 single‐blinded, placebo‐controlled studies with 27 Japanese and 50 Chinese participants were conducted. Osocimab was investigated with IV doses of 0.3, 1.25, and 2.5 mg/kg (Chinese study) and 0.3, 1.25, and 5.0 mg/kg (Japanese study), as well as SC doses of 3.0 and 6.0 mg/kg (Chinese study) and 6.0 mg/kg (Japanese study). The maximum plasma concentration was reached 1–3 h and 4–6 days after IV and SC administration, respectively. Osocimab exhibited a deviation from dose‐proportional pharmacokinetics for AUC but not *C*_max_; higher doses had higher apparent clearance and disproportionately lower total exposure. A slightly lower exposure was observed in Japanese compared with Chinese volunteers after IV administration; conversely, relatively higher exposure in Japanese volunteers with SC dosing was identified. Osocimab was associated with a dose‐dependent increase in activated partial thromboplastin time (aPTT). Maximal aPTT prolongations were observed 1–4 h and 2–6 days after IV and SC administration, respectively. Anti‐drug antibodies of low titer were detected in 1/9 (11.1%) Japanese volunteers administered placebo and 26/40 (65.0%) Chinese volunteers administered osocimab. Adverse events were reported in 8/18 (44.4%) Japanese and 28/40 (70.0%) Chinese volunteers who received osocimab, as well as in 1/9 (11.1%) Japanese and 6/10 (60.0%) Chinese volunteers who received placebo. In conclusion, data did not suggest a clear dose‐proportionality for osocimab within the investigated dose range. The effect of osocimab on aPTT was expected per its mechanism of action. Osocimab was generally well tolerated.

## Graphical abstract

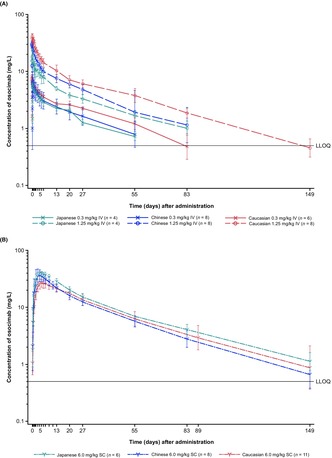

**Abbreviations**
aPTT: activated partial thromboplastin time
AUC: area under the plasma concentration–time curve from zero to infinity after single dose
*C*_max_
: maximum observed drug concentration in plasma after single‐dose administration
IV: intravenous
SC: subcutaneous

## 1 INTRODUCTION

Thromboembolic disorders are a major cause of disease and death globally, causing or contributing to acute coronary syndromes, embolic and thrombotic stroke, deep vein thrombosis, and pulmonary embolism.^1^, ^2^, ^3^ The reported incidence of venous thromboembolism has increased in recent decades in different populations, including in Japanese and Chinese populations.^4^

The use of factor Xa or thrombin inhibitors is associated with meaningful reductions in thrombotic events. However, there is a risk of bleeding with these current pharmacological approaches.^5^ There remains a clinical need for alternative treatment options for thrombotic events with an improved safety profile and an equivalent or superior efficacy profile compared with current treatment options.

The coagulation cascade comprises the contact pathway and the tissue factor pathway, which together mediate both thrombosis and hemostasis.^6^, ^7^ Factor XI (FXI) is the inert precursor form of activated FXI (FXIa)^8^; FXIa is essential for thrombin generation and thrombus formation.^7^, ^9^ However, evidence supports that hemostasis is predominantly mediated by the extrinsic and common coagulation pathways independently of FXI.^6^, ^9^, ^10^ In addition, studies of people with reduced levels of FXI and FXI deficiency (hemophilia type C) indicate a reduced incidence of venous thrombosis and stroke with a mild bleeding profile.^11^, ^12^, ^13^ Therefore, inhibition of FXI is hypothesized to reduce thrombosis while having a minimal effect on hemostasis.

Osocimab is a fully human immunoglobulin (Ig)G1 antibody that specifically blocks the catalytic activity of FXIa.^14^ Osocimab binds near the catalytic domain and thus modifies the recognition site of FXIa for proper binding of endogenous substrates.^14^ Consequently, this leads to blockade of the intrinsic activation and amplification loop of the coagulation cascade.^14^ The pharmacokinetics and pharmacodynamics of osocimab have been previously characterized in two phase 1 studies in healthy Caucasian volunteers (European Union Drug Regulating Authorities Clinical Trials Database [EudraCT]: 2017‐001937‐26 and 2014‐003816‐35).^15^ Osocimab had an acceptable safety profile and increased activated partial thromboplastin time (aPTT) in a dose‐dependent manner without bleeding reported.^15^

Here, results from two phase 1 studies in Japanese and Chinese healthy volunteers are reported. Collectively, these two studies evaluated the safety, tolerability, pharmacokinetics, and pharmacodynamic‐related parameters of different doses of intravenous (IV) and subcutaneous (SC) osocimab. In addition, the effect of ethnicity as an intrinsic factor (Japanese and Chinese [East Asian] vs. Caucasian) on the pharmacokinetics of osocimab is discussed.

## 2 METHODS

### 2.1 Study participants

Eligibility for the Japanese study included healthy male Japanese volunteers aged between ≥20 and ≤40 years, with a body weight ≤95 kg and a body mass index (BMI) ≥17.6 and ≤26.4 kg/m^2^. Eligibility for the Chinese study included healthy male Chinese volunteers aged between ≥20 and ≤45 years, with a body weight between ≥50 and ≤95 kg and a BMI ≥19 and <28 kg/m^2^.

### 2.2 Study designs

**FIGURE 1:**
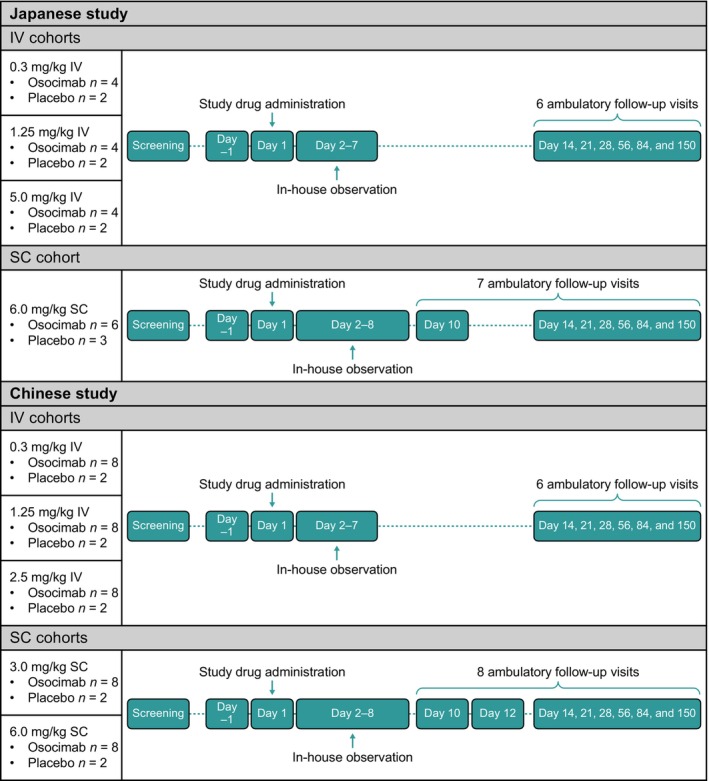
Overall design of studies.

Both studies had a randomized, single‐blinded, placebo‐controlled, single‐dose escalation design (Table 1). Eligible participants were sequentially assigned to a unique randomization number. The numbers were randomly assigned to study medication according to a computer‐generated randomization list. Participants were randomized to a single IV dose of 0.3, 1.25, and 2.5 mg/kg (Chinese study) and 0.3, 1.25, and 5.0 mg/kg (Japanese study), or a single SC dose of 3.0 and 6.0 mg/kg (Chinese study) and 6.0 mg/kg (Japanese study), or corresponding placebo control of each dose (Figure 1). Participants randomized to IV placebo were pooled for safety analysis in each study. Similarly, participants randomized to SC placebo were pooled for safety analysis in each study. Dose levels for IV and SC osocimab were informed by two previously completed studies in healthy Caucasian volunteers.^15^

**TABLE 1:** Design overview of the phase 1 studies in healthy Japanese and Chinese male participants.

Study ID	Population description	Study design	Osocimab dose and route of administration
17956	Healthy male Japanese participants	Single‐center, randomized, single‐blinded, placebo‐controlled, single‐dose escalation, sequential group phase 1 study	Three escalating IV dose levels of 0.3, 1.25, and 5.0 mg/kg administered as a 60‐min infusion, one SC dose level of 6.0 mg/kg
19355	Healthy male Chinese participants	Single‐center, randomized, single‐blinded, placebo‐controlled, single‐dose escalation, sequential group phase 1 study	Three escalating IV dose levels of 0.3, 1.25, and 2.5 mg/kg administered as a 60‐min infusion, two SC dose levels of 3.0 and 6.0 mg/kg

Abbreviations: ID, identification; IV, intravenous; SC, subcutaneous.

Osocimab was provided as a lyophilisate. The IV study medication was reconstituted and diluted with normal saline to a concentration of 0.2–20 mg/mL and administered as a single infusion over 60 min. The SC study medication was reconstituted with sterile water to 150 mg/mL and injected into SC abdominal fat. A dose of 6.0 mg/kg SC was given as two 3.0 mg/kg SC injections with a distance between them of at least 10 cm. Dose steps were enrolled sequentially; safety and tolerability were confirmed prior to dose escalation in both studies.

Both studies investigated the safety, pharmacokinetics, and immunogenicity associated with single doses of osocimab administered IV and SC. In addition, the Japanese study investigated aPTT as a pharmacodynamic parameter.

The Japanese study was conducted in Fukuoka Mirai Hospital, Fukuoka, Japan, and the Chinese study was conducted at the Drug Clinical Trial Centre, Peking University Third Hospital, Beijing, China. All participants provided written informed consent. Both studies met all local legal and regulatory requirements and were conducted in accordance with the ethical principles that have their origins from the Declaration of Helsinki and the International Conference on Harmonization Good Clinical Practice Guidelines.

### 2.3 Pharmacokinetics evaluation

Total plasma osocimab concentration was quantitatively determined in tripotassium ethylenediaminetetraacetic acid plasma using a biotinylated and Sulfo‐Tag‐labeled anti‐idiotypic antibody (bridging assay format).^15^ Osocimab controls, quality controls, and study samples were bound to biotinylated capture antibody‐coated streptavidin plates, followed by incubation with a Sulfo‐Tag‐labeled tracer antibody. A read buffer was added to create an appropriate chemical environment for electrochemiluminescence. Quantification of the antigen was performed using a Meso Scale Discovery instrument. The calibration range was from 0.500 mg/L (lower limit of quantitation [LLOQ]) to 6.00 mg/L. In the Japanese study, the mean inter‐accuracy of back‐calculated concentrations (except for LLOQ) ranged between 98.1% and 108%, and the precision was ≤3.73%. Accuracy and precision at the lowest calibrator (LLOQ) were 103% and 2.83%, respectively. Quality control samples in the concentration range of 1.25–5.00 mg/L had accuracy and precision ranges of 100%–109% and 6.13%–8.12%, respectively. In the Chinese study, the mean inter‐accuracy of back‐calculated concentrations (except for LLOQ) ranged between 99.2% and 103.6%, and the precision was ≤2.98%. Accuracy and precision at the lowest calibrator (LLOQ) were 100.9% and 10.5%, respectively. Quality control samples in the concentration range of 1.25–5.00 mg/L had accuracy and precision ranges of 95.2%–98.0% and 6.1%–7.9%, respectively.

Blood samples for pharmacokinetic analyses were collected predose and at prespecified time points up to 149 days post‐dose (Table S1). Pharmacokinetic parameters were calculated using a model‐independent, noncompartmental method. Analyses of pharmacokinetics evaluations were performed using Phoenix® WinNonlin® software (version 5.3 [Japanese study] or 8.1 [Chinese study], Certara L.P., Princeton, NJ, USA). Estimated pharmacokinetic parameters included area under the plasma concentration–time curve from zero to infinity after single dose (AUC), AUC from time zero to the last data point >LLOQ (AUC[0–*t*_last_]), total body clearance of drug from plasma calculated after IV administration (CL), maximum observed drug concentration in plasma after single‐dose administration (*C*_max_), time to reach *C*_max_ (*t*_max_), half‐life associated with the terminal slope (*t*_1/2_), and volume of distribution at steady state after intravascular administration (*V*_ss_).

### 2.4 aPTT evaluation

In the Japanese study, aPTT was assessed as a pharmacodynamic parameter via the kaolin‐trigger method, whereby citrated plasma was recalcified in the presence of a standardized quantity of cephalin (a platelet substitute) and kaolin (a factor XII activator).^15^ The assay was performed using a validated method with the STA compact analyzer (Diagnostica Stago). Results for aPTT were summarized as ratio to baseline over time. In the Chinese study, aPTT was measured as a safety parameter at the hospital's clinical laboratory. Blood samples for quantitative assessments of aPTT were collected predose and at prespecified time points up to 149 days post‐dose (Table S1). The aPTT values and changes are not to be quantitatively compared between the Japanese and Chinese studies due to methodological differences.

### 2.5 Immunogenicity evaluation

In both studies, the development of anti‐drug antibodies was assessed at a central laboratory. Binding and neutralizing anti‐drug antibodies were determined in the collected samples. Blood samples for anti‐drug antibodies were collected predose and at prespecified time points up to 149 days post‐dose (Table S1). Anti‐drug antibodies were determined in plasma after dilution with assay buffer by an ELISA with electrochemiluminescence readout using biotinylated osocimab as a capture and Sulfo‐tagged osocimab as a detection reagent. The assay sensitivity was not reduced at a concentration of osocimab up to 50.0 mg/L and was reduced by a factor of 2 at concentrations of osocimab >100 mg/L.

### 2.6 Safety assessments

The incidences of adverse events (AEs) were monitored and summarized by treatment group using the Medical Dictionary for Regulatory Activities (version 22.0 in the Japanese study and version 24.1 in the Chinese study). The study investigators classified the intensities of AEs as either mild, moderate, or severe. Events were defined as treatment‐emergent AEs (TEAEs) if they had started or worsened after single‐dose administration of study medication until the last visit, planned up to 149 days post‐dose. Infusion reactions and hypersensitivity were assessed according to the Common Terminology Criteria for Adverse Events (version 4.03 in both studies). Other safety assessments included laboratory assessments (hematology, blood chemistry, and clotting status) and 12‐lead electrocardiograms (ECG).

### 2.7 Statistical analyses

All safety, pharmacokinetics, aPTT, and immunogenicity results were summarized using descriptive statistics. Analyses of safety and anti‐drug antibodies were performed for all volunteers who received osocimab or placebo (safety analysis set). Volunteers who received osocimab and had evaluable pharmacokinetics data with no relevant validity findings for pharmacokinetics were included in the evaluation of pharmacokinetics (pharmacokinetics set).

To investigate dose proportionality, an analysis of variance (ANOVA; including the factor treatment) was performed for the Chinese study on the following log‐transformed pharmacokinetic parameters: AUC(0–*t*_last_)/D, AUC/D, and *C*_max_/*D* following IV and SC administration. Deviation from dose proportionality over the considered dose range was detected if the hypothesis “H: μ_1_=…=μ_s_ versus K: μ_i_≠μ_j_ for some i, j” was rejected by the *F*‐test at a significance level of *α* = .05, where μ_i_ is the expected logarithm of the pharmacokinetics parameter at the *i* ^th^ dose. “*s*” is the number of dose steps.

### 2.8 Nomenclature of targets and ligands

Key protein targets and ligands in this article are hyperlinked to corresponding entries in http://www.guidetopharmacology.org, the common portal for data from the IUPHAR/BPS Guide to PHARMACOLOGY,^16^ and are permanently archived in the Concise Guide to PHARMACOLOGY 2019/20.^17^

## 3 RESULTS

### 3.1 Demographics and characteristics at screening

Demographics at screening for both studies are summarized in Table 2. A total of 27 Japanese and 50 Chinese participants were enrolled and received study medication in the Japanese and Chinese studies, respectively. The Japanese and Chinese populations had mean ages of 27.4 and 31.0 years, BMIs of 21.6 and 23.9 kg/m^2^, and body weights of 62.9 and 70.8 kg at screening, respectively. Eighteen Japanese and 40 Chinese participants who received osocimab were included in the pharmacokinetics analysis. Within each study, demographics were comparable across all treatment groups and in participants included for pharmacokinetic analysis and safety analysis.

**TABLE 2:** Demographics at screening (safety analysis set).

	Osocimab						Placebo		Osocimab			Placebo	
	0.3 mg/kg IV		1.25 mg/kg IV		2.5 mg/kg IV	5.0 mg/kg IV	IV		3.0 mg/kg SC	6.0 mg/kg SC		SC	
	JPN (*n* = 4)	CHI (*n* = 8)	JPN (*n* = 4)	CHI (*n* = 8)	CHI (*n* = 8)	JPN (*n* = 4)	JPN (*n* = 6)	CHI (*n* = 6)	CHI (*n* = 8)	JPN (*n* = 6)	CHI (*n* = 8)	JPN (*n* = 3)	CHI (*n* = 4)
Male, *n* (%)	4 (100.0)	8 (100.0)	4 (100.0)	8 (100.0)	8 (100.0)	4 (100.0)	6 (100.0)	6 (100.0)	8 (100.0)	6 (100.0)	8 (100.0)	3 (100.0)	4 (100.0)
East Asian ethnicity, *n* (%)	4 (100.0)	8 (100.0)	4 (100.0)	8 (100.0)	8 (100.0)	4 (100.0)	6 (100.0)	6 (100.0)	8 (100.0)	6 (100.0)	8 (100.0)	3 (100.0)	4 (100.0)
Mean age, years (SD)	29.5 (6.1)	33.4 (4.6)	23.3 (3.2)	31.1 (5.1)	32.0 (6.4)	27.8 (4.1)	26.5 (3.9)	31.3 (6.1)	27.8 (3.2)	28.7 (4.5)	30.8 (6.5)	29.3 (3.2)	30.8 (5.4)
Mean body weight, kg (SD)	66.35 (5.49)	71.44 (2.60)	59.88 (6.36)	70.38 (4.72)	69.79 (8.37)	65.63 (9.36)	63.02 (6.07)	74.10 (4.76)	70.70 (6.91)	60.30 (6.62)	68.85 (7.06)	64.07 (3.32)	71.80 (2.92)
Mean height, cm (SD)	170.50 (2.38)	172.56 (4.25)	170.75 (3.77)	171.19 (1.87)	176.56 (7.28)	170.00 (5.48)	169.50 (5.75)	171.75 (9.01)	172.31 (4.87)	172.83 (3.19)	169.44 (4.38)	171.67 (3.79)	168.75 (3.50)
Mean BMI, kg/m^2^ (SD)	22.83 (1.91)	24.04 (1.61)	20.60 (2.56)	24.00 (1.27)	22.33 (2.02)	22.70 (2.80)	21.95 (2.17)	25.13 (1.15)	23.83 (2.24)	20.15 (1.55)	23.95 (1.96)	21.77 (1.48)	25.25 (1.49)
Smoking history, *n* (%)													
Current	1 (25.0)	1 (12.5)	1 (25.0)	0 (0.0)	1 (12.5)	2 (50.0)	1 (16.7)	0 (0.0)	0 (0.0)	0 (0.0)	1 (12.5)	0 (0.0)	0 (0.0)
Former	1 (25.0)	0 (0.0)	1 (25.0)	0 (0.0)	0 (0.0)	0 (0.0)	2 (33.3)	0 (0.0)	0 (0.0)	2 (33.3)	0 (0.0)	0 (0.0)	0 (0.0)
Never	2 (50.0)	7 (87.5)	2 (50.0)	8 (100.0)	7 (87.5)	2 (50.0)	3 (50.0)	6 (100.0)	8 (100.0)	4 (66.7)	7 (87.5)	3 (100.0)	4 (100.0)
Alcohol history, *n* (%)													
Non‐drinker	3 (75.0)	8 (100.0)	2 (50.0)	8 (100.0)	8 (100.0)	2 (50.0)	3 (50.0)	6 (100.0)	8 (100.0)	4 (66.7)	8 (100.0)	2 (66.7)	4 (100.0)
Light drinker	1 (25.0)	0 (0.0)	2 (50.0)	0 (0.0)	0 (0.0)	2 (50.0)	3 (50.0)	0 (0.0)	0 (0.0)	2 (33.3)	0 (0.0)	1 (33.3)	0 (0.0)

Abbreviations: BMI, body mass index; CHI, Chinese volunteers; IV, intravenous; JPN, Japanese volunteers; SC, subcutaneous; SD, standard deviation.

### 3.2 Pharmacokinetics evaluation

**FIGURE 2:**
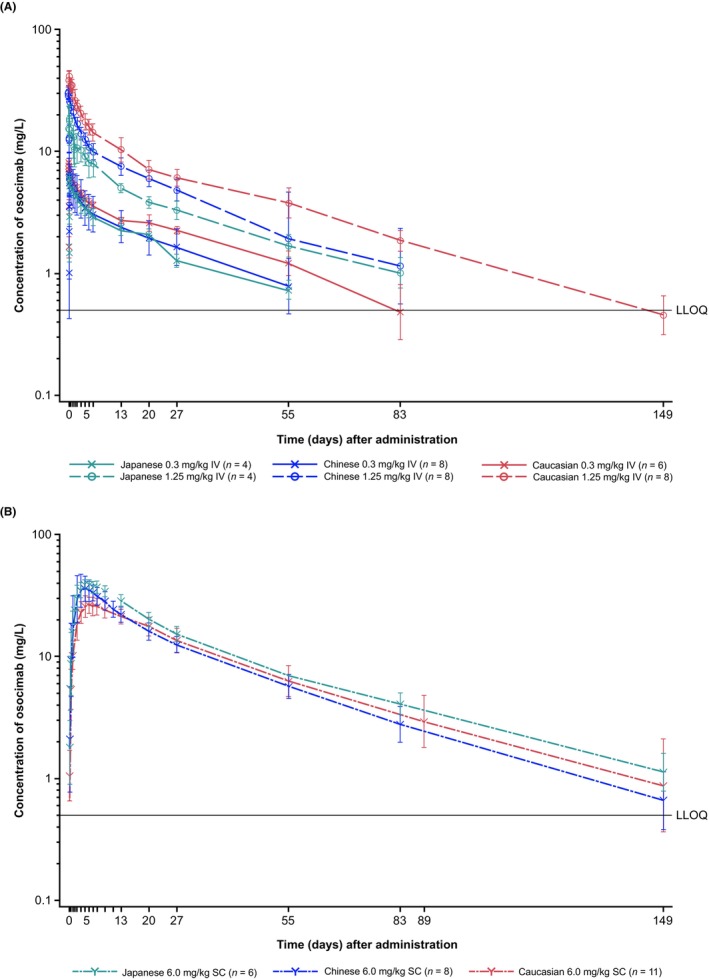
Geometric mean/standard deviation for concentrations of osocimab in plasma over 149 days in Chinese, Japanese, and Caucasian^a^ volunteers after intravenous (IV) dosing (A) and subcutaneous (SC) dosing (B). The lower limit of quantitation (LLOQ) was 0.5000 mg/L. ^a^EudraCT: 2014‐003816‐35 and 2017‐001937‐26.

Pharmacokinetics parameters are presented for both studies in Table 3. The concentration–time curves for the IV osocimab 0.3 and 1.25 mg/kg doses and SC osocimab 6.0 mg/kg dose are presented for both the Japanese and Chinese studies (Figure 2 [semi‐logarithmic scale] and Figure S1 [linear scale]).

**TABLE 3:** Summary of selected osocimab pharmacokinetic parameters following administration to Japanese and Chinese volunteers (pharmacokinetics set).

Parameter, unit	Osocimab								
	0.3 mg/kg IV		1.25 mg/kg IV		2.5 mg/kg IV	5.0 mg/kg IV	3.0 mg/kg SC	6.0 mg/kg SC	
	JPN (*n* = 4)	CHI (*n* = 8)	JPN (*n* = 4)	CHI (*n* = 8^a^)	CHI (*n* = 8)	JPN (*n* = 4)	CHI (*n* = 8)	JPN (*n* = 6)	CHI (*n* = 8)
AUC, mg·h L^−1^	2880 (14.6)	3390 (42.3)	7510 (20.3)	11 100 (25.2)	17 100 (23.2)	24 000 (9.41)	16 100 (26.1)	32 600 (11.2)	25 900 (17.8)
AUC(0–*t*_last_), mg·h L^−1^	2310 (13.4)	2640 (56.1)	6390 (17.1)	9940 (26.5)	16 100 (23.7)	23 100 (10.3)	15 200 (27.1)	31 100 (9.87)	24 700 (17.6)
AUC/D, h L^−1^	144 (6.64)	156 (42.1)	102 (16.2)	127 (31.3)	98.4 (16.9)	74.0 (20.7)	77.2 (30.8)	92.8 (18.8)	63.2 (24.2)
AUC(0–*t*_last_)/*D*, h L^−1^	116 (6.08)	121 (56.2)	87.2 (13.1)	113 (32.3)	92.6 (17.4)	71.2 (21.7)	72.6 (31.8)	88.7 (17.7)	60.2 (23.5)
*C*_max_, mg/L	5.90 (6.95)	6.91 (40.7)	19.9 (17.2)	30.9 (5.0)	52.0 (19.6)	91.8 (16.7)	18.0 (21.0)	39.4 (8.0)	38.2 (25.1)
*C*_max_/*D*, L^−1^	0.296 (6.14)	0.317 (41.2)	0.272 (21.1)	0.349 (8.4)	0.300 (19.2)	0.283 (12.2)	0.086 (26.1)	0.113 (13.9)	0.093 (32.0)
*t*_max_, h	2.00 (0.967–2.00)	3.00 (1.0–4.0)	1.48 (0.967–2.00)	1.00 (1.0–2.0)	1.50 (1.0–2.0)	2.48 (0.967–4.00)	132.00 (72.0−169.2)	108 (95.9− 168)	96.0 (48.0–216.9)
*t*_1/2_, h	573 (7.67)	696 (25.6)	783 (10.7)	774 (17.9)	773 (25.8)	720 (19.3)	713 (23.6)	853 (13.4)	725 (18.5)
CL, mL h^−1^	6.93 (6.64)	6.41 (42.1)	9.76 (16.2)	7.90 (31.3)	10.2 (16.9)	13.5 (20.7)	–	–	–
CL/*F*, mL h^−1^	–	–	–	–	–	–	12.9 (30.8)	10.8 (18.8)	15.8 (24.2)
*V*_ss_, *L*	5.50 (7.28)	6.08 (31.9)	9.60 (7.90)	7.76 (15.3)	9.08 (12.8)	10.3 (8.45)	–	–	–
*V*_z_/*F*, *L*	–	–	–	–	–	–	13.3 (11.8)	13.3 (13.6)	16.5 (29.5)

*Note:* Data shown as geometric means (geometric coefficient of variation %), except for *t*_max_, which is presented as the median (range). Abbreviations: AUC, area under the plasma concentration versus time curve from zero to infinity after a single dose; AUC/D, AUC divided by dose; AUC(0–*t*_last_), AUC from time 0 to the last data point >LLOQ, calculated up by linear trapezoidal rule, down by logarithmic trapezoidal rule; AUC(0–*t*_last_)/D, AUC(0–*t*_last_) divided by dose; CHI, Chinese volunteers; CL/F, total body clearance of drug from plasma calculated after extravascular administration (e.g., apparent subcutaneous clearance); *C*_max_, maximum observed drug concentration in plasma after single‐dose administration; *C*_max_/*D*, *C*_max_ divided by dose; IV, intravenous; JPN, Japanese volunteers; LLOQ, lower limit of quantitation; SC, subcutaneous; *t*_max_, time to reach *C*_max_; *t*_1/2_, half‐life associated with the terminal slope; *V*_ss_, volume of distribution at steady state after intravascular administration; *V*_Z_/*F*, apparent volume of distribution during terminal phase after extravascular administration.

^a^ Pharmacokinetics sample collection was not done on days 14, 21, and 28 for one of the volunteers. Therefore, only *C*_max_ (*C*_max_, *C*_max_/*D*) and *t*_max_ were reported for this volunteer.

The maximum plasma concentration was reached after 1–3 h and 4–6 days after IV and SC administration, respectively. The t_1/2_ was generally long, with geometric means by dose ranging from 24 to 36 days. Geometric mean CL values were small for IV osocimab (range 6.41–13.5 mL h^−1^) and tended to increase with increasing dose. Geometric mean *V*_ss_ values also increased with increasing dose (range 5.50–10.3 L). A similar trend of increasing apparent clearance and apparent volume of distribution with increasing doses of osocimab administered SC was observed in the Chinese population. Coefficients of variation were low to moderate with IV and SC administration for *C*_max_ and AUC, within the range of 5%–42% (Table 3).

Exposures (*C*_max_/*D* and AUC/D) in Japanese volunteers were slightly lower than exposures in Chinese volunteers in IV‐dose groups; however, the exposure ratios (Japanese volunteers/Chinese volunteers) were higher in the SC‐dose group (point estimates were 1.21 and 1.47 for *C*_max_/*D* and AUC/D, respectively) (Table 4). There was no apparent difference for both t_1/2_ and t_max_ values between Japanese and Chinese volunteers (Table 3).

**TABLE 4:** Summary of ANOVA comparing different ethnic groups for AUC/D and *C*_max_/*D* of osocimab in plasma.

	Osocimab dose	Interethnic ratio	Point estimate (LSM)	90% CI	
				Lower limit	Upper limit
AUC/D, h L^−1^	0.3 mg/kg IV	Japanese/Caucasian^a^	0.7719	0.5606	1.0629
		Chinese/Caucasian^a^	0.8334	0.6377	1.0892
		East Asian^b^/Caucasian^a^	0.8021	0.6228	1.0329
		Japanese/Chinese	0.9262	0.6837	1.2546
	1.25 mg/kg IV	Japanese/Caucasian^a^	0.6792	0.5277	0.8742
		Chinese/Caucasian^a^	0.8386	0.6775	1.0380
		East Asian^b^/Caucasian^a^	0.7547	0.6211	0.9170
		Japanese/Chinese	0.8099	0.6255	1.0487
	6.0 mg/kg SC	Japanese/Caucasian^a^	1.6325	1.3411	1.9872
		Chinese/Caucasian^a^	1.1113	0.9282	1.3305
		East Asian^b^/Caucasian^a^	1.3469	1.1514	1.5756
		Japanese/Chinese	1.4690	1.1917	1.8110
C_max_/D, L^−1^	0.3 mg/kg IV	Japanese/Caucasian^a^	0.8806	0.6425	1.2070
		Chinese/Caucasian^a^	0.9459	0.7266	1.2314
		East Asian^b^/Caucasian^a^	0.9127	0.7113	1.1710
		Japanese/Chinese	0.9310	0.6903	1.2556
	1.25 mg/kg IV	Japanese/Caucasian^a^	0.6417	0.5478	0.7517
		Chinese/Caucasian^a^	0.8219	0.7223	0.9352
		East Asian^b^/Caucasian^a^	0.7262	0.6436	0.8195
		Japanese/Chinese	0.7808	0.6665	0.9146
	6.0 mg/kg SC	Japanese/Caucasian^a^	1.9627	1.6164	2.3832
		Chinese/Caucasian^a^	1.6275	1.3625	1.9440
		East Asian^b^/Caucasian^a^	1.7873	1.5309	2.0865
		Japanese/Chinese	1.2060	0.9809	1.4827

Abbreviations: ANOVA, analysis of variance; AUC/D, area under the plasma concentration versus time curve from zero to infinity after a single dose divided by dose; CI, confidence interval; *C*_max_/*D*, maximum drug concentration in plasma after a single dose administration divided by dose; IV, intravenous; LSM, least‐squares mean; SC, subcutaneous.

^a^ EudraCT: 2014‐003816‐35 and 2017‐001937‐26.

^b^ East Asian comprises the Japanese and Chinese volunteers.

In the Chinese study, an exploratory ANOVA, which included selected dose‐normalized exposure parameters, did not suggest dose proportionality for IV cohorts with respect to AUC (Table S2). The geometric mean value of dose‐normalized AUCs (AUC/D and AUC[0‐*t*_last_]/*D*) in both studies decreased with increasing IV and also SC dose. In contrast, *C*_max_/*D* was comparable across all the IV and SC doses (Table 3).

### 3.3 aPTT assessments

In the Japanese study, IV administration of osocimab was associated with dose‐dependent increases of aPTT (Figure S2A). Mean aPTT increased instantly upon initiation of the IV infusion, with maximal aPTT prolongations at 2 h after initiation of infusion. The maximum ratios to baseline in mean aPTT for the IV osocimab 0.3, 1.25, and 5.0 mg/kg doses were 1.36 (corresponding to an absolute value of 44.2 s), 1.63 (corresponding to an absolute value of 51.5 s), and 1.96 (corresponding to an absolute value of 62.7 s), respectively. Prolongations in aPTT were constant after the end of the 1‐h IV infusion: up to 3 h with osocimab 0.3 mg/kg and up to 7 h with the higher IV doses. Mean aPTT returned to the reference range (24–33 s) by 55, 83, and >149 days after infusion of IV osocimab 0.3, 1.25, and 5.0 mg/kg doses, respectively. In contrast, aPTT increased along with the absorption of osocimab after 6.0 mg/kg SC administration, reaching a maximum ratio to baseline on Day 6 (1.61, corresponding to an absolute value of 55.2 s). Thereafter, prolongations in aPTT declined. Mean aPTT value returned to the reference range by >149 days after injection of SC osocimab.

In the Chinese study, IV administration of osocimab was also associated with dose‐dependent increases of aPTT (Figure S2B). Mean aPTT increased instantly upon initiation of the IV infusion, with maximal aPTT prolongations 1–4 h after initiation. Mean aPTT returned to the reference range (28–42 s) by 4 days after infusion of IV osocimab 0.3 mg/kg and 55 days for both the 1.25 and 2.5 mg/kg IV osocimab doses. In contrast, aPTT increased along with the absorption of osocimab after SC administration. aPTT seemed to increase more rapidly after injection of SC osocimab 6.0 mg/kg compared with the SC osocimab 3.0 mg/kg injection, reaching maximum elevation in 2–3 days; values were similar thereafter in both groups. The mean aPTT value returned to the reference range 55 days after injection of SC osocimab.

The correlation between aPTT and osocimab plasma concentration in both studies is displayed in Figure S3. aPTT prolonged with increasing osocimab concentration and a saturation effect was observed at higher concentrations.

### 3.4 Immunogenicity evaluation

In the Japanese study, a positive binding anti‐drug antibody was detected in 1/3 (33.3%) of volunteers receiving SC placebo 20 days after study drug administration (Table 5).

**TABLE 5:** Summary of positive anti‐drug antibodies response following single‐dose administration of osocimab (safety analysis set).

			Anti‐drug antibody^a^			
Treatment	Days after study drug administration	Total, *n*	Negative, *n* (%)	Positive, *n* (%)	Not tested, *n* (%)	Anti‐drug antibody titer, median (range)
Japanese volunteers	Anytime in the study	27	26 (96.3)	1 (3.7)	N/A	1.0 (N/A)
Placebo SC	20	3	2 (66.7)	1 (33.3)	0	1.0 (N/A)
	Anytime in this group	3	2 (66.7)	1 (33.3)	N/A	1.0 (N/A)
Chinese volunteers	Anytime in the study	50	24 (48.0)	26 (52.0)	N/A	1.3 (1.0–3.5)
Osocimab 1.25 mg/kg IV	13	8	2 (25.0)	5 (62.5)	1 (12.5)^b^	1.3 (1.0–1.8)
	27	8	1 (12.5)	6 (75.0)	1 (12.5)^b^	1.3 (1.0–1.6)
	Anytime in this group	8	2 (25.0)	6 (75.0)	N/A	1.3 (1.0–1.8)
Osocimab 2.5 mg/kg IV	13	8	4 (50.0)	4 (50.0)	0	1.0 (1.0–1.0)
	27	8	3 (37.5)	5 (62.5)	0	1.8 (1.0–2.5)
	55	8	3 (37.5)	5 (62.5)	0	1.3 (1.0–2.0)
	83	8	7 (87.5)	1 (12.5)	0	2.0 (N/A)
	149	8	7 (87.5)	1 (12.5)	0	3.5 (N/A)
	Anytime in this group	8	1 (12.5)	7 (87.5)	N/A	1.4 (1.0–3.5)
Osocimab 3.0 mg/kg SC	13	8	4 (50.0)	4 (50.0)	0	1.0 (1.0–1.5)
	27	8	2 (25.0)	6 (75.0)	0	1.1 (1.0–1.4)
	55	8	4 (50.0)	4 (50.0)	0	1.0 (1.0–1.3)
	83	8	7 (87.5)	1 (12.5)	0	1.0 (N/A)
	Anytime in this group	8	2 (25.0)	6 (75.0)	N/A	1.0 (1.0–1.5)
Osocimab 6.0 mg/kg SC	13	8	7 (87.5)	1 (12.5)	0	1.6 (N/A)
	27	8	5 (62.5)	3 (37.5)	0	1.4 (1.0–1.9)
	55	8	2 (25.0)	6 (75.0)	0	1.4 (1.0–2.3)
	83	8	7 (87.5)	1 (12.5)	0	1.4 (N/A)
	Anytime in this group	8	1 (12.5)	7 (87.5)	N/A	1.4 (1.0–2.3)

Abbreviations: COVID‐19, coronavirus 2019; IV, intravenous; N/A, not applicable; SC, subcutaneous.

^a^ No anti‐drug antibodies were detected at baseline. No volunteer had a positive neutralizing antibody result in either study population.

^b^ This participant could not return to the study site for anti‐drug antibody sampling due to lockdown during the COVID‐19 pandemic.

In the Chinese study, the formation of anti‐drug antibodies was detected in 26 volunteers, with 6/8 (75.0%), 7/8 (87.5%), 6/8 (75.0%), and 7/8 (87.5%) volunteers in the IV osocimab 1.25 and 2.5 mg/kg groups and SC osocimab 3.0 and 6.0 mg/kg groups, respectively. Anti‐drug antibodies were predominantly detected 13, 27, and 55 days after study drug administration (Table 5). No correlation between dose strength and the number of participants with confirmed anti‐drug antibody formation could be identified. The titer for anti‐drug antibodies was low (overall range 1–3.5).

No anti‐drug antibodies were detected at baseline, and neutralizing antibody formation was negative in all volunteers (Table S3).

### 3.5 Safety assessments

In the Japanese study, TEAEs were observed in one of nine volunteers (11.1%) receiving placebo and eight of 18 (44.4%) volunteers receiving osocimab (Table 6). All reported TEAEs were of mild intensity, and there were no serious AEs (SAEs) or study discontinuations related to a TEAE. One volunteer who received SC osocimab 6.0 mg/kg had erythema, which was considered related to the study drug. Another volunteer had injection‐site erythema in the same group, which was considered not related to the study drug but related to the study procedures. The most commonly reported TEAEs were nasopharyngitis (in one volunteer receiving placebo and three receiving osocimab), increased C‐reactive protein (in three volunteers receiving osocimab), and increased blood creatinine phosphokinase (in two volunteers receiving osocimab). Except for aPTT, there was no evidence for laboratory parameter changes induced by administration of osocimab independent of the dose administered.

**TABLE 6:** Overall summary of the number and frequency of subjects with TEAEs (safety analysis set).

*n* (%)	Osocimab						Placebo		Osocimab			Placebo	
	0.3 mg/kg IV		1.25 mg/kg IV		2.5 mg/kg IV	5.0 mg/kg IV	Placebo IV		3.0 mg/kg SC	6.0 mg/kg SC		SC	
	JPN (*n* = 4)	CHI (*n* = 8)	JPN (*n* = 4)	CHI (*n* = 8)	CHI (*n* = 8)	JPN (*n* = 4)	JPN (*n* = 6)	CHI (*n* = 6)	CHI (*n* = 8)	JPN (*n* = 6)	CHI (*n* = 8)	JPN (*n* = 3)	CHI (*n* = 4)
Any AE	2 (50.0)	5 (62.5)	1 (25.0)	6 (75.0)	4 (50.0)	2 (50.0)	1 (16.7)	4 (66.7)	6 (75.0)	3 (50.0)	7 (87.5)	0	2 (50.0)
Any study drug‐related AE	0	0	0	0	2 (25.0)	0	0	0	1 (12.5)	1 (16.7)	1 (12.5)	0	0
Any AE related to procedures	0	0	0	0	0	0	0	0	0	1 (16.7)	0	0	0
Maximum intensity													
Mild	2 (50.0)	5 (62.5)	1 (25.0)	6 (75.0)	4 (50.0)	2 (50.0)	1 (16.7)	4 (66.7)	6 (75.0)	3 (50.0)	6 (75.0)	0	2 (50.0)
Severe	0	0	0	0	0	0	0	0	0	0	1 (12.5)	0	0
Maximum intensity for study drug‐related AEs													
Mild	0	0	0	0	2 (25.0)	0	0	0	1 (12.5)	1 (16.7)	0	0	0
Severe	0	0	0	0	0	0	0	0	0	0	1 (12.5)	0	0
AE‐related deaths	0	0	0	0	0	0	0	0	0	0	0	0	0
Any SAE	0	0	0	0	0	0	0	0	0	0	1 (12.5)	0	0
Any study drug‐related SAE	0	0	0	0	0	0	0	0	0	0	1 (12.5)	0	0
Discontinuation of study drug due to AEs	0	0	0	0	0	0	0	0	0	0	0	0	0

Abbreviations: AE, adverse event; CHI, Chinese volunteers; IV, intravenous; JPN, Japanese volunteers; SAE, serious adverse event; SC, subcutaneous; TEAE, treatment‐emergent adverse event.

In the Chinese study, TEAEs were observed in six of 10 (60.0%) volunteers receiving placebo and 28 of 40 (70.0%) volunteers receiving osocimab (Table 6). All reported TEAEs were of mild intensity, except for one severe thrombocytopenia event in a volunteer after receiving SC osocimab 6.0 mg/kg. This volunteer experienced platelet decrease within 48 h. Their platelet level decreased from a baseline of 183 × 10^9^/L (shortly before drug administration) to 83 × 10^9^/L (reference range: 125–350 × 10^9^/L). Platelet level continued to decrease to a nadir of 13 × 10^9^/L in another 48 h. This severe thrombocytopenia event was classified as an SAE due to requiring hospitalization to a hematology ward for further assessment and treatment and was considered to be related to the study drug. No bleeding or any other symptoms, including bruising or petechiae, were reported/observed during physical examination. The volunteer was asked to stay in bed and received IV Ig and fresh‐frozen plasma. His platelet level returned to the reference range 7 days after the nadir and remained around the baseline level during the rest of the study. The volunteer also had skin exfoliation on both hands 21 days after study drug administration, which was resolved with urea ointment. The study participant did not report any sequelae. Besides the severe thrombocytopenia and skin exfoliation, three mild TEAEs in three other volunteers were considered to be related to the study drug (two receiving IV osocimab 2.5 mg/kg and one receiving SC osocimab 3.0 mg/kg; thrombocytopenia, injection‐site erythema, and epistaxis in one volunteer each). Other commonly reported TEAEs were upper respiratory tract infection (two volunteers receiving placebo and eight receiving osocimab) and increased blood triglycerides (one volunteer receiving placebo and seven receiving osocimab). None were considered to be related to the study drug. Except for the single severe thrombocytopenia event and the expected changes in aPTT, osocimab had no relevant effect on laboratory parameters.

In both studies, osocimab was not associated with clinically relevant effects on vital signs or ECG.

## 4 DISCUSSION

Osocimab was characterized in two phase 1 studies enrolling healthy Japanese and Chinese volunteers. Osocimab has been previously studied in healthy Caucasian populations in both IV and SC preparations (EudraCT: 2014‐003816‐35 and 2017‐001937‐26). To further contextualize the results in Japanese and Chinese volunteers, an exploratory ANOVA, which included the factor “ethnicity” as a fixed effect, was performed per dose level for AUC/D and *C*_max_/*D*. Based on these analyses, point estimates (LSM) and corresponding exploratory 90% CIs for the interethnic ratios (Japanese/Caucasian, Chinese/Caucasian, and pooled East Asian/Caucasian) were estimated for the osocimab 0.3 and 1.25 mg/kg IV doses and the 6.0 mg/kg SC dose (Table 4). In general, for the IV osocimab groups, the pooled East Asian participant group showed no consistent trend in exposure when compared to Caucasian participants. The exposure of the SC osocimab 6.0 mg/kg group in East Asian volunteers was slightly higher than that in Caucasian volunteers. However, the interethnic ratios are based on a low sample size and are purely exploratory.

To date, there have been no clinically significant pharmacokinetic differences reported between East Asian and Caucasian healthy volunteers, according to research results on IgG1 antibodies.^18^, ^19^, ^20^ Furthermore, IgG antibodies have a high selectivity profile with their target site; as a result, interaction with off‐target sites is unlikely.^20^ Owing to their large molecular weight, polarity, and hydrophilicity, IgG antibodies are primarily distributed in the intercellular junctions of the vascular endothelium, with a limited and slow distribution to peripheral tissue.^20^ Therefore, there seem to be few concerns regarding off‐target effects.^20^

In both studies, aPTT prolonged with increasing osocimab plasma concentration, which was consistent with its mechanism of action. The course of aPTT changes was similar in Japanese and Chinese volunteers. Quantitatively, the maximum effect on aPTT in the first 28 days after osocimab administration, that is, maximum ratio to baseline, was compared between Japanese and Caucasian volunteers for the osocimab 0.3, 1.25, and 5 mg/kg IV doses and the 6.0 mg/kg SC dose using an ANCOVA per dose level with the factor ethnicity as a fixed effect and baseline as a covariate. The maximum aPTT ratio to baseline in Japanese volunteers was 92.4% of that in Caucasian volunteers (90% CI: 87.7%–97.3%) for 0.3 mg/kg IV. For the 1.25 mg/kg IV, 5 mg/kg IV, and 6 mg/kg SC groups, the maximum effect in the first 28 days was similar in Japanese and Caucasian volunteers, with interethnic ratios close to unity and the 90% CIs included unity (Table S4).

Osocimab was generally well tolerated, with no clinically relevant effects on volunteers' vital signs or ECG readings. One SAE of severe thrombocytopenia was reported in the Chinese study. As the study was conducted in healthy volunteers, any underlying disease and treatment that may cause thrombocytopenia could be ruled out. After detecting the critically low level of platelets in this instance, a blood smear confirmed the result and showed no platelet aggregation. Direct activity by osocimab on platelets was unlikely, as an in vitro study demonstrated no specific binding to resting or activated platelets (data on file). Considering the acute drop in platelet level, antibody‐mediated platelet destruction should be suspected.^21^ However, such antibodies are unlikely to have been induced by osocimab. The decrease in platelet level was observed 48 h after drug administration, and it takes 3–4 days of antigen exposure in secondary lymphoid tissue germinal centers (GC) for B cells to integrate input from cytokines and T‐cells for activation of certain transcription factors to dictate differentiation into antibody‐producing plasma cells, GC B cells, or memory B cells.^22^ At the time of the studies, osocimab was an investigational new drug and a new substance to the volunteer; therefore, an antibody is unlikely to have been produced by memory B cells either. The anti‐drug antibody results also support this point: at the nadir of platelet level, the anti‐drug antibody result was negative. A low titer of anti‐drug antibodies was detected 2 weeks after drug administration, when the platelet level had already returned to the reference range. Even though a low titer of anti‐drug antibodies was still detectable 4 and 8 weeks (titers of 1.9 and 1.0, respectively) after drug administration, the platelet level was normal for the rest of the study. This volunteer also had skin exfoliation on both hands from 3 to 6 weeks after drug administration, when anti‐drug antibodies were detectable. Skin exfoliation may represent one of the immune response patterns.^23^ However, the lesions were only restricted to both hands, could be treated with urea ointment, and did not recur even in the presence of anti‐drug antibodies. Therefore, it was uncertain whether the skin exfoliation of this volunteer was related to an immune response against osocimab. No further investigations were carried out to identify the causative mechanism.

The Japanese and Chinese studies enrolled healthy volunteers, and the results may not necessarily reflect what may be observed in a population with disease. However, the tolerability and efficacy of osocimab have been reported for patients in two phase 2 studies.^24^, ^25^, ^26^ While there were some differences between the designs of the Japanese and Chinese studies, the collection of samples related to pharmacokinetics, aPTT, and anti‐drug antibodies was similar and at similar time points.

In conclusion, data did not suggest a clear dose‐proportionality for osocimab within the investigated dose range. The effect of osocimab on aPTT was expected per its mechanism of action and correlated with the osocimab concentrations in plasma. In addition, osocimab was generally well tolerated. The titer of anti‐drug antibodies was low, though the incidence was high in Chinese volunteers. No positive neutralizing antibody was detected. Differences in the pharmacokinetics of osocimab were partly observed between East Asian and Caucasian volunteers at an exploratory level; however, there is no clinically meaningful difference in the pharmacodynamics and safety of osocimab between the East Asian and Caucasian populations. Therefore, no adjustments to osocimab dosing recommendations are warranted for future clinical studies.

## AUTHOR CONTRIBUTIONS

All authors substantially contributed to the conception or design of the studies, or the acquisition, analysis, or interpretation of data. All authors drafted the work and/or reviewed it critically for important intellectual content.

## FUNDING INFORMATION

Funding for this research was provided by Bayer AG Berlin, Germany.

## CONFLICT OF INTEREST STATEMENT

Frauke Friedrichs is an employee of Bayer AG. Kensei Hashizume and Toshiaki Tanaka are employees of Bayer Yakuhin, Ltd, and may own stock in the company. Zhili Dong, Pei Liu, and Yuqin Liao are employees of Bayer Healthcare Company Ltd.

## ETHICS STATEMENT

Both studies met all local legal and regulatory requirements and were conducted in accordance with the ethical principles that have their origins from the Declaration of Helsinki and the International Conference on Harmonization Good Clinical Practice Guidelines.

## PATIENT CONSENT STATEMENT

All participants provided written informed consent.

## Supporting information


**Data S1:** Supporting Information.

## Data Availability

Availability of the data underlying this publication will be determined according to Bayer's commitment to the EFPIA/PhRMA “Principles for responsible clinical trial data sharing.” This pertains to scope, timepoint, and process of data access. As such, Bayer commits to sharing upon request from qualified scientific and medical researchers, patient‐level clinical trial data, study‐level clinical trial data, and protocols from clinical trials in patients for medicines and indications approved in the United States (US) and European Union (EU) as necessary for conducting legitimate research. This applies to data on new medicines and indications that have been approved by the EU and US regulatory agencies on or after January 01, 2014. Interested researchers can use www.vivli.org to request access to anonymized patient‐level data and supporting documents from clinical studies to conduct further research that can help advance medical science or improve patient care. Information on the Bayer criteria for listing studies and other relevant information is provided in the member section of the portal. Data access will be granted to anonymized patient‐level data, protocols, and clinical study reports after approval by an independent scientific review panel. Bayer is not involved in the decisions made by the independent review panel. Bayer will take all necessary measures to ensure that patient privacy is safeguarded.
