# Supplementary material for: Pharmacokinetics and pharmacodynamics of the factor XIa‐inhibiting antibody osocimab in healthy male East Asian volunteers: Results from two phase 1 studies
Source: Pharmacol Res Perspect. 2024 Sep 22;12(5):e70012. doi: 10.1002/prp2.70012 (PMC11417140; doi:10.1002/prp2.70012)
Supplement: Supplementary file 1 — Data S1: Supporting Information. [file PRP2-12-e70012-s001.docx]

# Supplementary materials

TABLE S1 Blood PK sampling design

| **Study day** | **0** | | | | | | | | | | **1** | | **2** | **3** | **4** | **5** | **6** | **7** | **9** | **11** | **13** | **20** | **27** | **55** | **83** | **149** |
| --- | --- | --- | --- | --- | --- | --- | --- | --- | --- | --- | --- | --- | --- | --- | --- | --- | --- | --- | --- | --- | --- | --- | --- | --- | --- | --- |
| **Hour(s)** | **−0** | **0** | **0.25** | **0.5** | **0.75** | **1** | **2** | **4** | **8** | **12** | **24** | **36** | **48** | **72** | **96** | **120** | **144** | **-** | **-** | **-** | **-** | **-** | **-** | **-** | **-** | **-** |
| Administration of study drug | - | X^a^🡪 | 🡪 | 🡪 | 🡪 | 🡪 | - | - | - | - | - | - | - | - | - | - | - | - | - | - | - | - | - | - | - | - |
| PK sampling | X^b^ | - | X^c^ | X^c^ | - | X | X^c^ | X | X^d^ | X | X | X^d^ | X | X | X | X | X | X^e^ | X^e^ | X^e,f^ | X | X | X | X | X | X |
| Sampling for aPTT | X^b^ | - | - | - | - | X | X^d^ | X | X^d^ | X | X | X^d^ | X | X | X | X | X | X^e^ | X^e^ | X^e,f^ | X | X | X | X | X | X |
| ADA | X^b^ | - | - | - | - | - | - | - | - | - | - | - | - | - | - | - | X^g^ | - | - | - | X | X^g^ | X | X | X | X |

^a^Only for SC osocimab. Arrows indicate osocimab IV administered as a single infusion over 60 min.
^b^PK, PD and ADA sampling at pre-dose.
^c^Only for IV osocimab.
^d^In the Chinese study, sampling was for the IV cohorts only.
^e^Sampling for the SC cohorts only.
^f^Sampling in the Chinese study only.
^g^Samping in the Japanese study only.
Abbreviations: ADA, anti-drug antibody; aPTT, activated partial thromboplastin time; IV, intravenous; SC, subcutaneous; PD, pharmacodynamic; PK, pharmacokinetic.

TABLE S2 Summary of ANOVA on logarithms of pharmacokinetic parameters following study drug administration to Chinese volunteers (pharmacokinetics set)

| **Parameter, unit** | **Degrees of freedom** | **Mean sum of squares** | **F-value** | ***P*-value** |
| --- | --- | --- | --- | --- |
| **IV cohort** | | | | |
| AUC(0–t_last_)/D, h L^−1^ | 2 | 0.154 | 1.13 | 0.343 |
| AUC/D, h L^−1^ | 2 | 0.425 | 4.47 | 0.0248 |
| C_max_/D, L^−1^ | 2 | 0.047 | 0.70 | 0.507 |
| **SC cohort** | | | | |
| AUC(0–t_last_)/D, h L^−1^ | 1 | 0.141 | 1.88 | 0.192 |
| AUC/D, h L^−1^ | 1 | 0.161 | 2.18 | 0.162 |
| C_max_/D, L^−1^ | 1 | 0.024 | 0.29 | 0.596 |

Abbreviations: ANOVA, analysis of variance; AUC, area under the plasma concentration versus time curve from zero to infinity after single (first) dose; AUC/D, AUC divided by dose; AUC(0–t_last_), AUC from time 0 to the last data point >LLOQ, calculated up by linear trapezoidal rule, down by logarithmic trapezoidal rule; AUC(0–t_last_)/D, AUC(0–t_last_) divided by dose; C_max_, maximum observed drug concentration in plasma after single dose administration; C_max_/D, C_max_ divided by dose; IV, intravenous; LLOQ, lower limit of quantitation; SC, subcutaneous.

TABLE S3 Number and percentage of volunteers who developed anti-drug antibodies (safety analysis set)

| **Treatment** | **Time point** | **Total,  *n*** | **Negative,  *n* (%)** | | **Positive,  *n* (%)** | **Not done,  *n* (%)** |
| --- | --- | --- | --- | --- | --- | --- |
| **Japanese volunteers** | | | |  | | |
| Osocimab 0.3 mg/kg IV | Day 0 (baseline) | 4 | 4 (100.0) | | 0 | 0 |
|  | Day 6 | 4 | 4 (100.0) | | 0 | 0 |
|  | Day 13 | 4 | 4 (100.0) | | 0 | 0 |
|  | Day 20 | 4 | 4 (100.0) | | 0 | 0 |
|  | Day 27 | 4 | 4 (100.0) | | 0 | 0 |
|  | Day 55 | 4 | 4 (100.0) | | 0 | 0 |
|  | Day 83 | 4 | 4 (100.0) | | 0 | 0 |
|  | Day 149 | 4 | 4 (100.0) | | 0 | 0 |
| Osocimab 1.25 mg/kg IV | Day 0 (baseline) | 4 | 4 (100.0) | | 0 | 0 |
|  | Day 6 | 4 | 4 (100.0) | | 0 | 0 |
|  | Day 13 | 4 | 4 (100.0) | | 0 | 0 |
|  | Day 20 | 4 | 4 (100.0) | | 0 | 0 |
|  | Day 27 | 4 | 4 (100.0) | | 0 | 0 |
|  | Day 55 | 4 | 4 (100.0) | | 0 | 0 |
|  | Day 83 | 4 | 4 (100.0) | | 0 | 0 |
|  | Day 149 | 4 | 4 (100.0) | | 0 | 0 |
| Osocimab 5.0 mg/kg IV | Day 0 (baseline) | 4 | 4 (100.0) | | 0 | 0 |
|  | Day 6 | 4 | 4 (100.0) | | 0 | 0 |
|  | Day 13 | 4 | 4 (100.0) | | 0 | 0 |
|  | Day 20 | 4 | 4 (100.0) | | 0 | 0 |
|  | Day 27 | 4 | 4 (100.0) | | 0 | 0 |
|  | Day 55 | 4 | 4 (100.0) | | 0 | 0 |
|  | Day 83 | 4 | 4 (100.0) | | 0 | 0 |
|  | Day 149 | 4 | 4 (100.0) | | 0 | 0 |
| Placebo IV | Day 0 (baseline) | 6 | 6 (100.0) | | 0 | 0 |
|  | Day 6 | 6 | 6 (100.0) | | 0 | 0 |
|  | Day 13 | 6 | 6 (100.0) | | 0 | 0 |
|  | Day 20 | 6 | 6 (100.0) | | 0 | 0 |
|  | Day 27 | 6 | 6 (100.0) | | 0 | 0 |
|  | Day 55 | 6 | 6 (100.0) | | 0 | 0 |
|  | Day 83 | 6 | 6 (100.0) | | 0 | 0 |
|  | Day 149 | 6 | 6 (100.0) | | 0 | 0 |
| Osocimab 6.0 mg/kg SC | Day 0 (baseline) | 6 | 6 (100.0) | | 0 | 0 |
|  | Day 6 | 6 | 6 (100.0) | | 0 | 0 |
|  | Day 13 | 6 | 6 (100.0) | | 0 | 0 |
|  | Day 20 | 6 | 6 (100.0) | | 0 | 0 |
|  | Day 27 | 6 | 6 (100.0) | | 0 | 0 |
|  | Day 55 | 6 | 6 (100.0) | | 0 | 0 |
|  | Day 83 | 6 | 6 (100.0) | | 0 | 0 |
|  | Day 149 | 6 | 6 (100.0) | | 0 | 0 |
| Placebo SC | Day 0 (baseline) | 3 | 3 (100.0) | | 0 | 0 |
|  | Day 6 | 3 | 3 (100.0) | | 0 | 0 |
|  | Day 13 | 3 | 3 (100.0) | | 0 | 0 |
|  | Day 20 | 3 | 2 (66.7) | | 1 (33.3) | 0 |
|  | Day 27 | 3 | 3 (100.0) | | 0 | 0 |
|  | Day 55 | 3 | 3 (100.0) | | 0 | 0 |
|  | Day 83 | 3 | 3 (100.0) | | 0 | 0 |
|  | Day 149 | 3 | 3 (100.0) | | 0 | 0 |
| **Chinese volunteers** | | | | | | |
| Osocimab 0.3 mg/kg IV | Day 0 (baseline) | 8 | 8 (100.0) | | 0 | 0 |
|  | Day 13 | 8 | 8 (100.0) | | 0 | 0 |
|  | Day 27 | 8 | 8 (100.0) | | 0 | 0 |
|  | Day 55 | 8 | 8 (100.0) | | 0 | 0 |
|  | Day 83 | 8 | 8 (100.0) | | 0 | 0 |
|  | Day 149 | 8 | 8 (100.0) | | 0 | 0 |
| Osocimab 1.25 mg/kg IV | Day 0 (baseline) | 8 | 8 (100.0) | | 0 | 0 |
|  | Day 13 | 8 | 2 (25.0) | | 5 (62.5) | 1 (12.5) |
|  | Day 27 | 8 | 1 (12.5) | | 6 (75.0) | 1 (12.5) |
|  | Day 55 | 8 | 8 (100.0) | | 0 | 0 |
|  | Day 83 | 8 | 8 (100.0) | | 0 | 0 |
|  | Day 149 | 8 | 8 (100.0) | | 0 | 0 |
| Osocimab 2.5 mg/kg IV | Day 0 (baseline) | 8 | 8 (100.0) | | 0 | 0 |
|  | Day 13 | 8 | 4 (50.0) | | 4 (50.0) | 0 |
|  | Day 27 | 8 | 3 (37.5) | | 5 (62.5) | 0 |
|  | Day 55 | 8 | 3 (37.5) | | 5 (62.5) | 0 |
|  | Day 83 | 8 | 7 (87.5) | | 1 (12.5) | 0 |
|  | Day 149 | 8 | 7 (87.5) | | 1 (12.5) | 0 |
| Placebo IV | Baseline | 6 | 6 (100.0) | | 0 | 0 |
|  | Day 13 | 6 | 6 (100.0) | | 0 | 0 |
|  | Day 27 | 6 | 6 (100.0) | | 0 | 0 |
|  | Day 55 | 6 | 6 (100.0) | | 0 | 0 |
|  | Day 83 | 6 | 6 (100.0) | | 0 | 0 |
|  | Day 149 | 6 | 6 (100.0) | | 0 | 0 |
| Osocimab 3.0 mg/kg SC | Day 0 (baseline) | 8 | 8 (100.0) | | 0 | 0 |
|  | Day 13 | 8 | 4 (50.0) | | 4 (50.0) | 0 |
|  | Day 27 | 8 | 2 (25.0) | | 6 (75.0) | 0 |
|  | Day 55 | 8 | 4 (50.0) | | 4 (50.0) | 0 |
|  | Day 83 | 8 | 7 (87.5) | | 1 (12.5) | 0 |
|  | Day 149 | 8 | 8 (100.0) | | 0 | 0 |
| Osocimab 6.0 mg/kg SC | Day 0 (baseline) | 8 | 8 (100.0) | | 0 | 0 |
|  | Control measurement 1 at baseline | 1 | 1 (100.0) | | 0 | 0 |
|  | Day 13 | 8 | 7 (87.5) | | 1 (12.5) | 0 |
|  | Day 27 | 8 | 5 (62.5) | | 3 (37.5) | 0 |
|  | Day 55 | 8 | 2 (25.0) | | 6 (75.0) | 0 |
|  | Day 83 | 8 | 7 (87.5) | | 1 (12.5) | 0 |
|  | Day 149 | 8 | 7 (87.5) | | 0 | 1 (12.5) |
| Placebo SC | Day 0 (baseline) | 4 | 4 (100.0) | | 0 | 0 |
|  | Day 13 | 4 | 4 (100.0) | | 0 | 0 |
|  | Day 27 | 4 | 4 (100.0) | | 0 | 0 |
|  | Day 55 | 4 | 4 (100.0) | | 0 | 0 |
|  | Day 83 | 4 | 4 (100.0) | | 0 | 0 |
|  | Day 149 | 4 | 4 (100.0) | | 0 | 0 |

Abbreviations: IV, intravenous; SC, subcutaneous.

TABLE S4 Summary of ANCOVA for aPTT^a^ maximum ratio to baseline in first 28 days after dosing in the Japanese and Caucasian studies

|  | | | **90% CI** | |
| --- | --- | --- | --- | --- |
| **Treatment** | **Interethnic ratio** | **Point estimate**  **(LSM)** | **Lower limit** | **Upper limit** |
| 0.3 mg/kg IV | Japanese/Caucasian | 0.924 | 0.877 | 0.973 |
| 1.25 mg/kg IV | Japanese/Caucasian | 0.978 | 0.889 | 1.08 |
| 5 mg/kg IV | Japanese/Caucasian | 1.02 | 0.973 | 1.06 |
| 6 mg/kg SC | Japanese/Caucasian | 1.05 | 0.999 | 1.10 |

^a^In the Japanese and the Caucasian studies, aPTT was assessed as a pharmacodynamic parameter via the kaolin-trigger method.

Abbreviations: ANCOVA, analysis of covariance; aPTT, activated partial thromboplastin time; CI, confidence interval; IV, intravenous; LSM, least-squares mean; SC, subcutaneous.

FIGURE S1 Geometric mean/standard deviation for concentration of IV osocimab 0.3 and 1.25 mg/kg (A) and SC osocimab 6.0 mg/kg (B) in plasma over 149 days in Chinese, Japanese and Caucasian^a^ volunteers


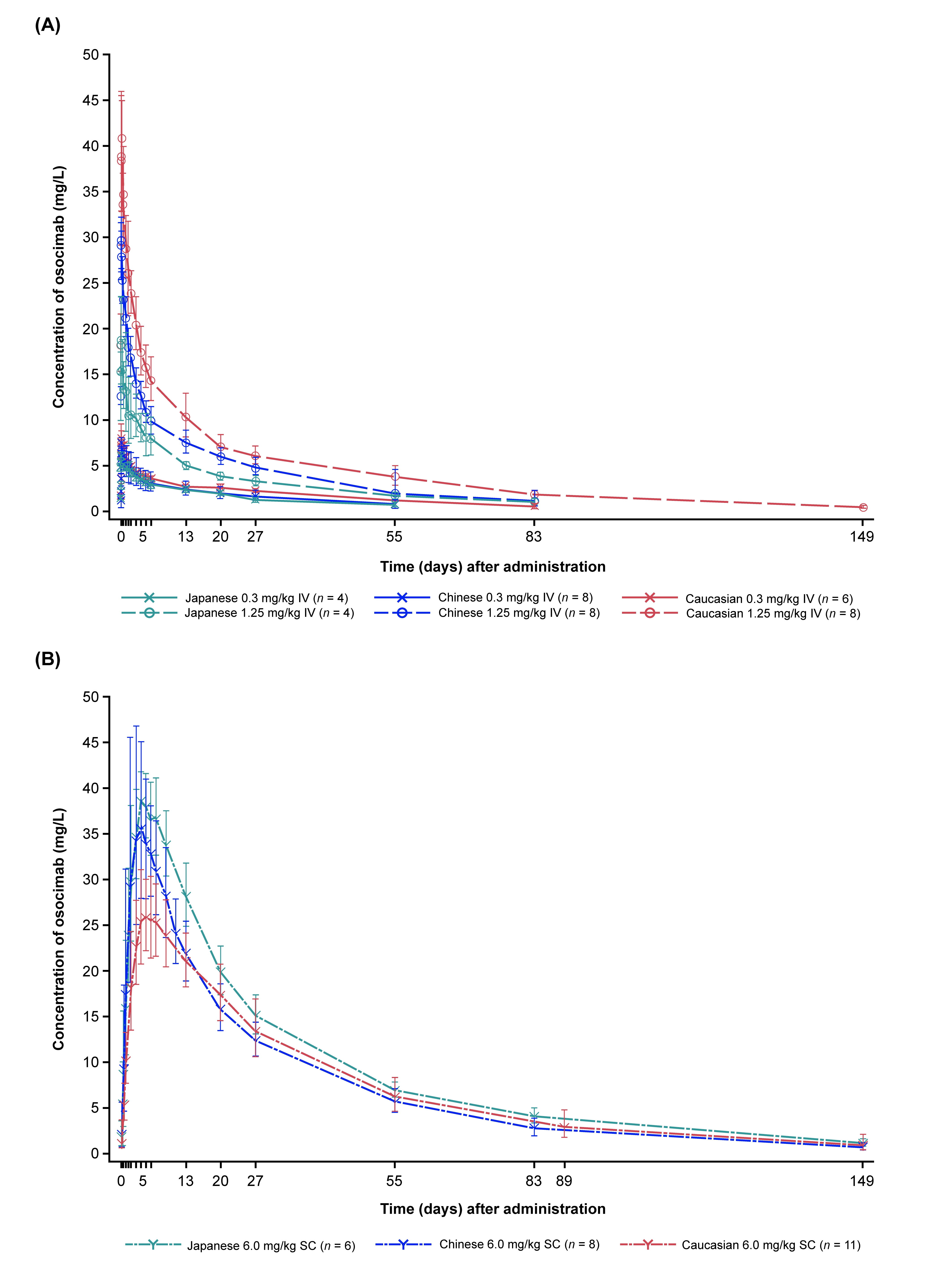


IV, intravenous; LLOQ, lower limit of quantification; SC, subcutaneous.
^a^EudraCT: 2014-003816-35 and 2017-001937-26.

FIGURE S2 Mean and standard deviation for aPTT over 149 days in the Japanese study^a^ (A) and the Chinese study^b^ (B)


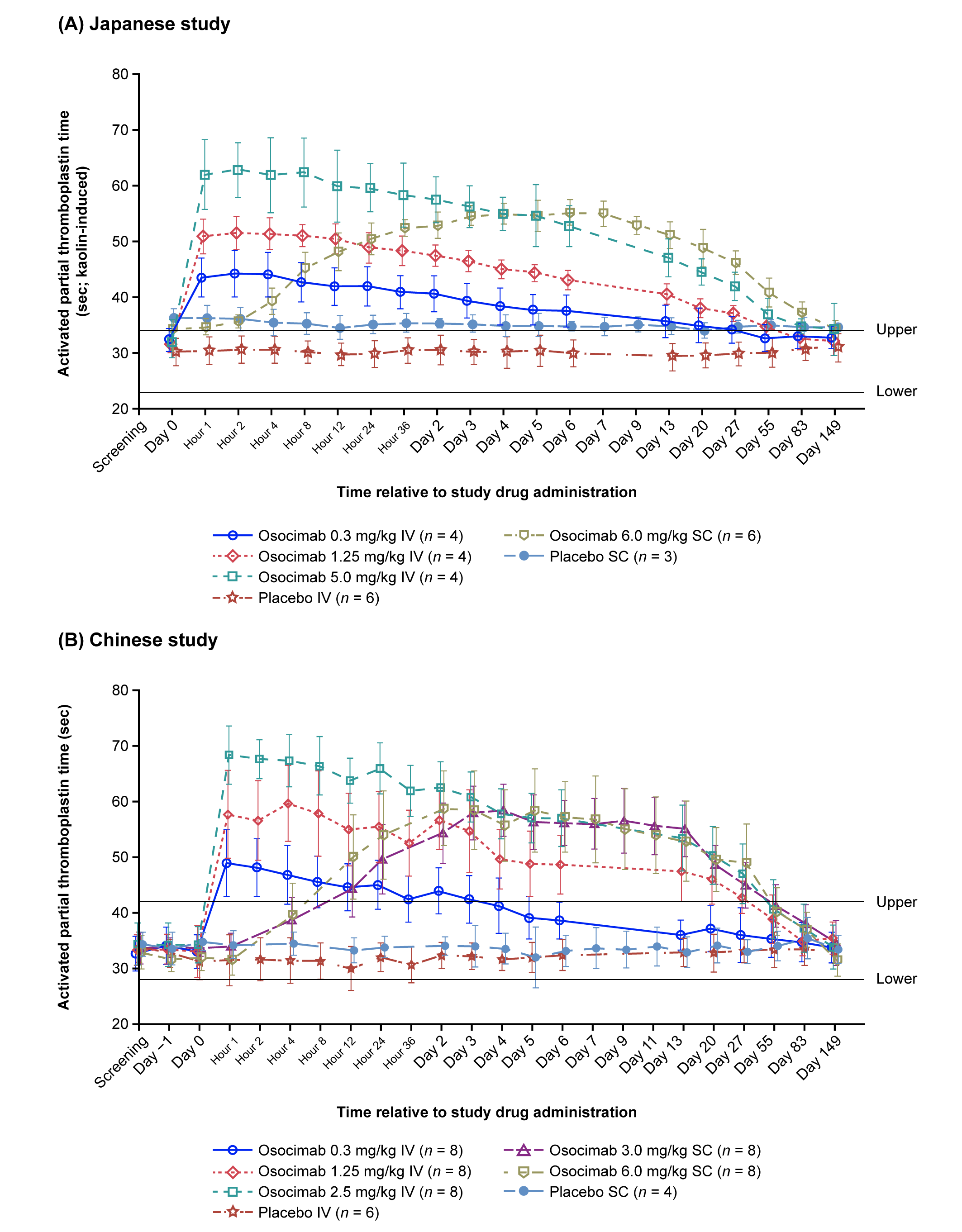


aPTT, activated partial thromboplastin time; IV, intravenous; SC, subcutaneous.
Upper and lower lines refer to the reference range for measurement of aPTT; 24–33 and 28–42 seconds in the Japanese and Chinese studies, respectively.
^a^In the Japanese study, aPTT was assessed as a pharmacodynamic parameter via the kaolin-trigger method.
^b^In the Chinese study, aPTT was measured as a safety parameter at the hospital’s clinical laboratory.

FIGURE S3 Correlation of aPTT and osocimab concentration in plasma in the Japanese study^a^ (A) and the Chinese study^b^ (B)


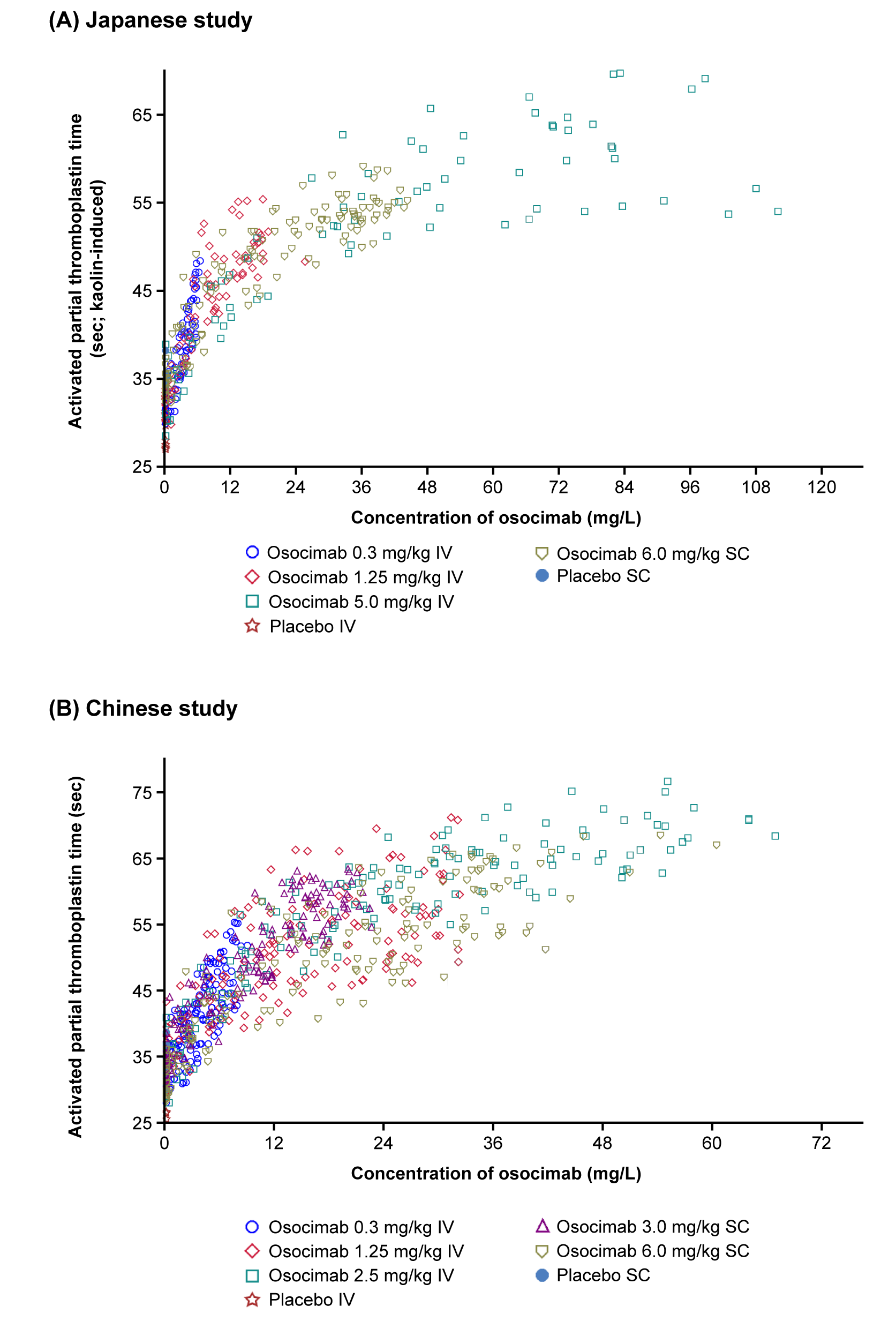


aPTT, activated partial thromboplastin time.
^a^In the Japanese study, aPTT was assessed as a pharmacodynamic parameter via the kaolin-trigger method.
^b^In the Chinese study, aPTT was measured as a safety parameter at the hospital’s clinical laboratory.
